# The gut–heart axis in cardio-oncology

**DOI:** 10.1093/cvr/cvag146

**Published:** 2026-06-30

**Authors:** Nicole S L Chan, Courtney Cross, Joanne Bowen, Clive Prestidge, Feargal J Ryan, Mohsen Dorraki, Sanuri Liyanage, Shiwani Sharma, Rajesh Pathy, Christina A Bursill, Peter J Psaltis, Francine Z Marques, Hannah R Wardill, Paul Joyce

**Affiliations:** Translational Nanomedicine & Biotherapeutics Group, School of Pharmacy and Biomedical Science, Adelaide University, Adelaide City West Campus, North Terrace, Adelaide, SA 5000, Australia; School of Pharmacy and Biomedical Science, College of Health, Adelaide University, Adelaide, SA 5000, Australia; Supportive Oncology Research Group, Precision Cancer Medicine Theme, South Australian Health and Medical Research Institute (SAHMRI), Adelaide, SA 5000, Australia; School of Pharmacy and Biomedical Science, College of Health, Adelaide University, Adelaide, SA 5000, Australia; School of Pharmacy and Biomedical Science, College of Health, Adelaide University, Adelaide, SA 5000, Australia; Flinders Health and Medical Research Institute, Flinders University, Bedford Park, SA, Australia; Precision Cancer Medicine Theme, South Australian Health and Medical Research Institute (SAHMRI), Adelaide, SA 5000, Australia; School of Computer Science and Information Science, Adelaide University, Adelaide, SA 5005, Australia; Australian Institute for Machine Learning (AIML), Adelaide University, Adelaide, SA 5000, Australia; Vascular, Research Centre, Lifelong Health Theme, South Australian Health and Medical Research Institute (SAHMRI), Adelaide, SA 5000, Australia; Vascular, Research Centre, Lifelong Health Theme, South Australian Health and Medical Research Institute (SAHMRI), Adelaide, SA 5000, Australia; Vascular, Research Centre, Lifelong Health Theme, South Australian Health and Medical Research Institute (SAHMRI), Adelaide, SA 5000, Australia; Vascular, Research Centre, Lifelong Health Theme, South Australian Health and Medical Research Institute (SAHMRI), Adelaide, SA 5000, Australia; School of Medicine, College of Health, Adelaide University, Adelaide, SA 5000, Australia; Vascular, Research Centre, Lifelong Health Theme, South Australian Health and Medical Research Institute (SAHMRI), Adelaide, SA 5000, Australia; School of Medicine, College of Health, Adelaide University, Adelaide, SA 5000, Australia; Department of Cardiology, Central Adelaide Local Health Network, Adelaide, SA 5000, Australia; Hypertension Research Laboratory, Department of Pharmacology, Biomedical Discovery Institute, Monash University, Melbourne, Australia; Victorian Heart Institute, Monash University, Melbourne, Australia; Heart Failure Research Group, Baker Heart and Diabetes Institute, Melbourne, VIC 3004, Australia; School of Pharmacy and Biomedical Science, College of Health, Adelaide University, Adelaide, SA 5000, Australia; Supportive Oncology Research Group, Precision Cancer Medicine Theme, South Australian Health and Medical Research Institute (SAHMRI), Adelaide, SA 5000, Australia; Translational Nanomedicine & Biotherapeutics Group, School of Pharmacy and Biomedical Science, Adelaide University, Adelaide City West Campus, North Terrace, Adelaide, SA 5000, Australia

**Keywords:** Chemotherapy-induced cardiotoxicity, Cardiovascular health, Microbiota, Immunotherapy, Radiotherapy, Cancer therapy-related cardiac dysfunction, CTRCD

## Abstract

As cancer survival rates improve, the long-term burden of treatment has become increasingly evident. Cancer survivors face a markedly higher risk of cardiovascular-related complications and premature, non–cancer-related mortality. In particular, cardiovascular disease (CVD) is disproportionately prevalent, with survivors approximately 40% more likely to develop and die from CVD compared to the general population. Although this increased morbidity reflects both acute cardiotoxic events and the later development and progression of more chronic CVDs, traditionally viewed as direct consequences of cancer therapies, the underlying mechanisms, especially those that are feasible to modify, are poorly understood. Emerging evidence positions the gut–heart axis as a central regulator of cardiovascular risk in cardio-oncology. This avenue is especially compelling to explore as the gut microbiome is well documented to be altered by cancer therapies, including chemotherapy, immune checkpoint inhibitors, targeted therapies, and radiation, with profound and persistent changes in diversity, composition and function widely reported across clinical cohorts. Comparable microbial changes have been observed in non-cancer cohorts with cardiovascular disease. For example, specific microbial metabolites have been reported to exert cardiovascular protective benefits in hypertension. Thus, there is a compelling opportunity to explore the gut microbiome to advance our understanding and ability to prevent cardiovascular disease in cancer survivors. This review synthesizes current evidence linking the gut microbiome to cancer therapy-related cardiac dysfunction (CTRCD), evaluates microbial metabolites as predictive biomarkers of cardiotoxicity, and discusses microbiome-targeted modulation as an emerging strategy for improving cardiovascular outcomes in cancer survivors.

## 1. Introduction

Advances in cancer therapy have significantly improved survival; however, therapeutic efficacy is increasingly offset by the burden of treatment-related toxicities, shifting focus towards long-term health outcomes in cancer patients and survivors. Among these, cancer therapy-related cardiac dysfunction (CTRCD) has emerged as one of the most serious and life-limiting complications.^1^ CTRCD can cause irreversible cardiac and vascular injury, substantially increasing long-term cardiovascular disease (CVD) risk among cancer survivors, who experience a 40% greater incidence of CVD and CVD-related mortality compared to those without a cancer history.^2–4^ Consequently, CVD now represents the second leading cause of death in cancer survivors, underscoring the need to clarify the underlying mechanisms driving chemotherapy-related cardiovascular complications to develop targeted preventive and therapeutic strategies.^4,5^

Modern oncology encompasses a diverse range of therapeutic modalities, including chemotherapy, immune checkpoint inhibitors (ICIs), targeted therapies, and radiation. While these treatments are integral to cancer care, each has been associated with distinct and sometimes overlapping cardiovascular toxicities. CTRCD therefore represents a broad clinical spectrum arising from the off-target effects of cancer therapies on metabolically active or proliferative non-malignant cardiovascular tissues, including cardiac myocytes and vascular endothelium.^1,6^ These toxicities may present acutely during or immediately after therapy, or manifest years later as progressive cardiovascular dysfunction, reflecting the complex and multifactorial nature of cardiotoxicity.^7^ Despite its growing clinical importance, the underlying pathophysiology of CTRCD remains incompletely understood, and current management strategies are limited. A further, critical unmet need in cardio-oncology is understanding the sources of interindividual variability in cardiovascular outcomes. Patients receiving similar treatments often exhibit markedly different cardiovascular trajectories, suggesting the presence of upstream modulators of cardiovascular susceptibility.

The gut microbiome has emerged as a key candidate in this regard, acting as a dynamic interface between host metabolism, immune signalling, and vascular biology. Specifically, the gut–heart axis, a bidirectional network linking the gut microbiome, its metabolites, and the cardiovascular system,^8–15^ may modulate cardiovascular risk across the entire cardio-oncology continuum, from baseline susceptibility to treatment-induced injury and long-term survivorship.^16^ Cancer therapies disrupt microbial composition and intestinal barrier integrity, promoting the systemic translocation of microbial products, such as lipopolysaccharides (LPS), and altering the production of bioactive metabolites, including trimethylamine-N-oxide (TMAO).^17^ Both LPS and TMAO are recognized contributors to chronic inflammation, immune activation, and vascular dysfunction and have been independently implicated in the pathogenesis of CVD.^18,19^ In contrast, gut-derived short-chain fatty acids (SCFAs) exert anti-inflammatory and cardioprotective effects through modulation of immune cell function, endothelial signalling, and metabolic homeostasis.^20,21^ Disruption of the balance between pro-inflammatory mediators (LPS, TMAO) and protective metabolites (SCFAs) may therefore represent a key mechanism through which cancer therapies exacerbate systemic inflammation and cardiovascular injury.^22^

As the role of the gut microbiome and its metabolite-driven inflammatory signalling in CTRCD remains poorly defined, a deeper understanding of these gut–heart interactions may yield novel mechanistic insights and therapeutic opportunities. This review, therefore, aims to position the gut–heart axis as a central mechanistic framework in cardio-oncology, critically evaluating current evidence linking the gut microbiome and cardiovascular outcomes across cancer therapies. A particular emphasis is placed on the role of chemotherapy in CTRCD, given this treatment modality has been the cornerstone of cancer care for several decades and therefore provides the most evidence linking the gut–heart axis to CTRCD. However, emerging evidence has also highlighted the capacity for immunotherapies, targeted therapies, and radiation to disrupt the gut microbiome and induce cardiovascular complications, suggesting there is a broader role of the gut–heart axis across all cardio-oncology. Insights derived from chemotherapy studies may also offer important conceptual and mechanistic parallels that extend to other cancer therapies, where evidence is rapidly evolving but remains comparatively limited. This review therefore provides a comprehensive overview of the current cardio-oncology landscape while seeking to identify critical knowledge gaps and emerging avenues for prevention, risk stratification, and intervention.

## 2. CTRCD: overview of mechanisms and clinical insights

The incidence, severity, and temporal presentation of CTRCD vary substantially depending on treatment modality, cumulative exposure, and patient-specific susceptibility factors.^23^ Across therapies, manifestations range from subclinical myocardial injury to overt heart failure, vascular events, arrhythmias, and inflammatory cardiac syndromes.^6^  *Table 1* provides a detailed overview of the known cancer therapies to induce cardiovascular complications, their proposed pathophysiological mechanisms, and key clinical insights. While chemotherapy remains the most extensively characterized, emerging therapies are increasingly implicated in clinically significant CTRCD. Notably, despite mechanistic heterogeneity, many therapies converge on shared downstream pathways, including oxidative stress, endothelial dysfunction, and inflammation.

**Table 1:** Cancer therapies associated with cardiovascular toxicities

Therapy class	Examples	Common cancer indications	Cardiovascular implications	Proposed mechanisms	Clinical Notes	Refs
Anthracyclines	DOX Idarubicin Daunorubicin	Breast cancer Haematological malignancies (leukaemia, lymphoma) Sarcomas Ovarian cancer	Direct cardiomyocyte injury Dilated cardiomyopathy Heart failure LV dysfunction Arrhythmias	ROS generation → oxidative stress Mitochondrial dysfunction Inhibition of TOP2β → DNA damage	Most studied class for cardiotoxicity Dose-dependent toxicity (≥400–550 mg/m^2^) Effects often irreversible Chronic and progressive in nature	^24–30^
Antimetabolites	5-FU Capecitabine Gemcitabine	Colorectal cancer Gastric cancer Breast cancer Pancreatic cancer Head and neck cancers	Vascular injury Coronary vasospasm Angina-like symptoms Arrhythmias Myocardial ischaemia/infarction	Endothelial dysfunction via ↓eNOS and NO PKC-mediated vasoconstriction Direct vascular toxicity Oxidative stress	Second most common agent in chemotherapy-induced cardiotoxicity Typically, functional and reversible High recurrence risk upon re-exposure	^31–35^
Alkylating agents	Cyclophosphamide Ifosfamide Bleomycin	Haematological malignancies (leukaemia, lymphoma) Breast cancer Testicular cancer	Myocarditis Tachyarrhythmias Heart failure (often irreversible)	Endothelial injury Oxidative stress Direct cardiomyocyte toxicity	Cardiotoxicity often occurs within days Dose dependent (e.g. >150 mg/kg for cyclophosphamide) Early symptoms may be non-specific	^36–39^
Platinum agents	Cisplatin Oxaliplatin	Testicular cancer Colorectal cancer Ovarian cancer Lung cancer	Cardiac ischaemia Myocardial infarction Chronic heart failure	Oxidative stress Endothelial dysfunction Inflammation	Cardiotoxicity may present acutely or be delayed Uncommon, but clinically significant toxicity Mechanisms still not fully understood	^40–43^
HER2-targeted therapies	Trastuzumab Pertuzumab	HER2 + breast cancer	LV dysfunction Heart failure	Inhibition of HER2 signalling → impaired cardiomyocyte survival pathways	Risk increases with concurrent treatment modalities Often reversible with cessation Frequent LVEF monitoring required	^44,45^
VEGF pathway inhibitors	Bevacizumab Sunitinib Sorafenib	Renal cancer Colorectal cancer Hepatocellular carcinoma	Hypertension Thrombosis Heart failure	Endothelial dysfunction ↓eNOS and NO	Hypertension occurs in nearly 100% of cases when VEGFIs are combined Requires regular blood pressure monitoring	^46,47^
Immune checkpoint inhibitors	Nivolumab Pembrolizumab Ipilimumab	Melanoma Lung cancer	Myocarditis Arrhythmias Pericarditis Heart failure	T-cell activation Immune-mediated inflammation	Rare but high mortality Early detection critical Limited associations	^48,49^
Cytokine-directed therapies	Tocilizumab (IL-6) CAR-T-associated CRS therapies	Haematological malignancies	Cardiomyopathy Heart failure Arrhythmias Myocardial infarction LV dysfunction	Immune-cytokine signalling dysregulation Endothelial dysfunction	Often acute and reversible Linked to CRS severity	^50^
Radiation therapy	Thoracic radiotherapy	Breast cancer Lymphoma Lung cancer	Coronary artery disease Myocardial fibrosis Cardiomyopathy Valvular disease Pericardial disease	Endothelial injury Chronic inflammation Fibrosis Microvascular damage	Delayed onset (years–decades) Dose and field dependent	^51^

DOX, doxorubicin; 5-FU, 5-fluorouracil; LV, left ventricular; ROS, reactive oxygen species; TOP2β, topoisomerase 2 beta; eNOS, endothelial nitric oxide synthase; NO, nitric oxide; PKC, protein kinase C; LVEF, left ventricular ejection fraction; VEGF, vascular endothelial growth factor; CRS, cytokine release syndrome.

Among chemotherapeutic agents, anthracyclines remain the archetypal model of dose-dependent cardiotoxicity, associated with left ventricular (LV) dysfunction and heart failure, with incidence rates approaching 48% at higher cumulative doses.^52^ In contrast, antimetabolites, such as 5-fluorouracil (5-FU), predominantly induce vascular toxicity, coronary vasospasm, and ischaemia, occurring in 2–5% of patients.^24,53^ Importantly, contemporary therapies, including ICIs, targeted therapies, and radiation, have expanded the spectrum of cardiovascular injury, introducing immune-mediated, hypertensive, and fibrotic phenotypes that may differ substantially in onset and reversibility.^44^

Beyond myocardial dysfunction, cancer therapies exert systemic vascular effects, contributing to endothelial dysfunction, thrombosis, hypertension, and pericardial disease.^6^ In a cohort study of 17 389 patients with breast, prostate, or colorectal cancer, more than half developed a cardiovascular complication within 12 months of completing cancer therapy, which was associated with a substantially increased risk of mortality risk in the subsequent year (56.42%).^54^ Despite this burden, current diagnostic approaches remain insufficiently sensitive to detect early subclinical injury, highlighting the importance of complementary mechanistic approaches, including emerging microbiome-targeted strategies.^55^

## 3. Pathophysiological mechanisms linking CTRCD to the gut–heart axis

**Figure 1:**
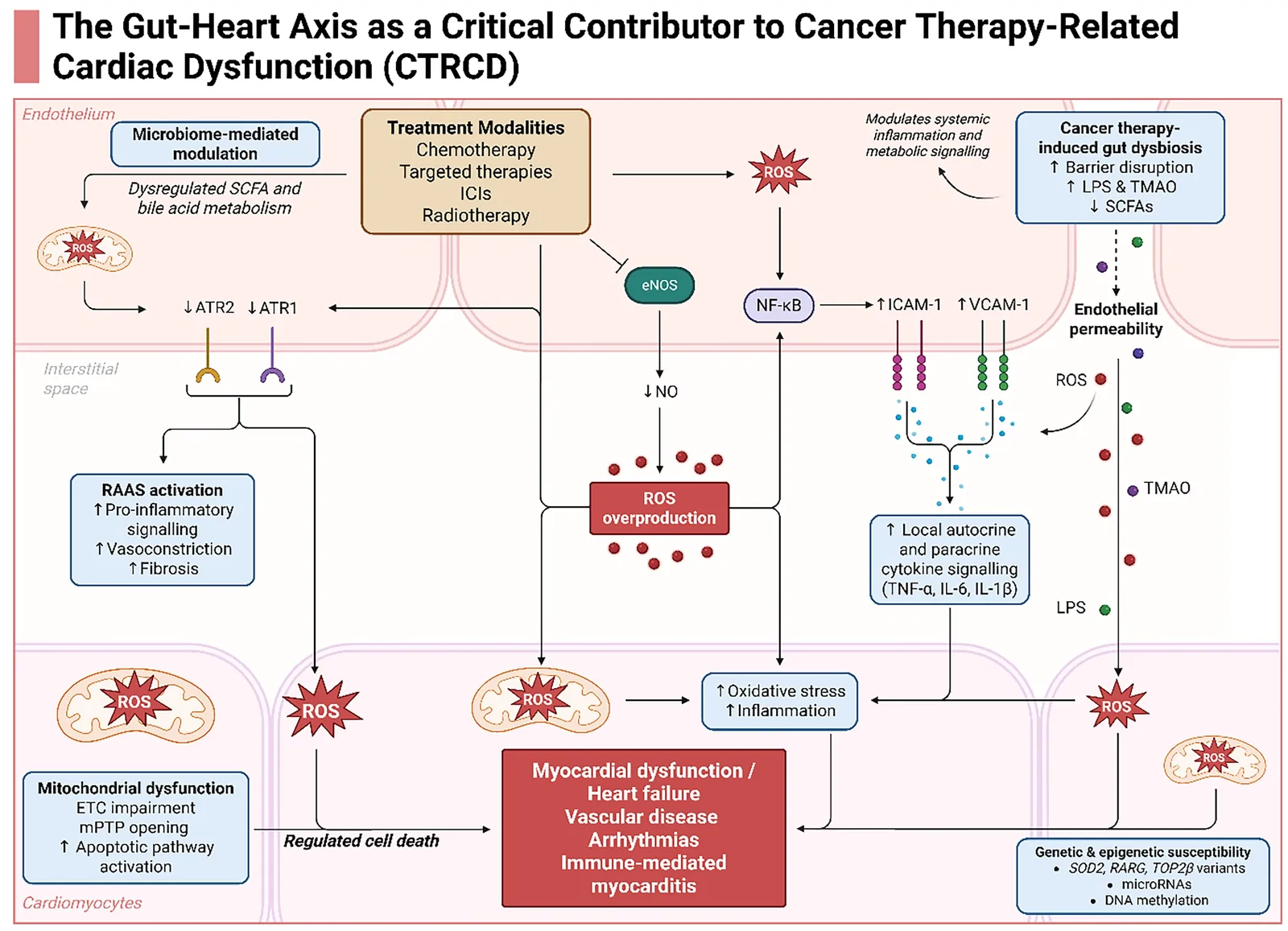
The gut–heart axis as a critical contributor to CTRCD. Cancer therapies, including chemotherapy, targeted therapies, immunotherapies, and radiotherapy, promote ROS production, activating convergent pathways, involving mitochondrial dysfunction, oxidative stress, inflammatory signalling, and endothelial injury. These processes collectively promote cardiomyocyte damage and vascular dysfunction, leading to diverse clinical phenotypes. Critically, these pathophysiological mechanisms are associated with cancer therapy-induced gut microbiome disruption, which depletes short-chain fatty acid production and promotes barrier dysfunction, leading to systemic translocation of endotoxins, such as LPS and TMAO into circulation, thereby further amplifying oxidative stress and inflammation. Genetic and epigenetic susceptibility factors (e.g. SOD2, RARG, TOP2β variants, and microRNA regulation) may further modulate these responses. Collectively, mitochondrial dysfunction, oxidative stress, and inflammatory signalling, processes that may be amplified by ATR1 and ATR2 receptor signalling, drive cardiomyocyte injury, regulated cell death pathways, and endothelial dysfunction, contributing to long-term cardiovascular complications following chemotherapy.

CTRCD arises from interconnected mechanisms involving oxidative stress, mitochondrial dysfunction, inflammatory signalling, endothelial injury, and regulated cell death,^56^ all factors associated with gut microbiome dysbiosis (*Figure 1*). These mechanisms underpin the heterogeneous clinical phenotypes of cardiotoxicity, consistent with recent European Society of Cardiology (ESC) cardio-oncology guidelines, including myocardial dysfunction, vascular toxicity, arrhythmias, and immune-mediated myocarditis, reflecting the diverse mechanisms through which anticancer therapies affect the cardiovascular system.^57,58^ Importantly, these processes do not operate in isolation but instead reflect a coordinated multi-system response to therapy-induced cellular stress. Within this framework, CTRCD can be conceptualized as a hierarchical cascade in which cancer therapy-induced disturbances are integrated across metabolic, immune, and vascular domains, ultimately converging on cardiomyocyte death. While these mechanisms are well established, therapy-induced disruption of the gut microbiome has emerged as a potential upstream modifier. Microbiome-derived metabolites, impaired barrier function, and altered host–microbiome interactions may constitute additional mechanistic layers that may influence the severity, persistence, and systemic propagation of cardiotoxic injury.

Although individual therapies may preferentially activate distinct upstream pathways, cytotoxic agents predominantly induce oxidative and mitochondrial injury, targeted therapies disrupt vascular and angiogenic signalling, immunotherapies drive immune-mediated inflammation, and radiation promotes chronic fibrotic and vascular remodelling.^59^ Host–microbiome interactions, alongside genetic and epigenetic susceptibility, modulate the severity and expression of these injury pathways.^56,60,61^ Cancer therapies disrupt gut microbial homeostasis through direct or indirect mechanisms. Certain agents, most notably anthracyclines, possess intrinsic antibiotic activity that directly disrupts gut microbial communities.^62^ In contrast, treatment-induced mucosal injury and compromised epithelial integrity^63,64^ can lead to nutrient depletion, oxygenation changes, and heightened immune activation, factors that indirectly create a selective niche favouring virulent, inflammation-associated pathogens while suppressing the growth of obligate anaerobic SCFA-producing commensals.^65^

### 3.1 Oxidative stress and mitochondrial dysfunction

Mitochondrial dysfunction represents a central mechanism in CTRCD, particularly in chemotherapy-associated injury. Given that mitochondria comprise approximately 30% of cardiomyocyte volume, cardiac tissue is particularly vulnerable to mitochondrial injury.^66^ Cancer therapies, including anthracyclines, platinum agents, and radiation, disrupt mitochondrial function, driving excessive reactive oxygen species (ROS) production and electron transport chain impairment.^56,60^ Excess ROS promotes mitochondrial DNA damage, calcium dysregulation, impairment of sirtuin-mediated metabolic regulation, and activation of pro-apoptotic signalling cascades.^60,67,68^ In parallel, mitochondrial dysfunction in vascular endothelial cells (ECs) and smooth muscle cells (SMCs) impairs redox balance, reduces nitric oxide (NO) bioavailability, and disrupts vascular tone, thereby promoting vascular inflammation and atherogenesis.^69^

Individual susceptibility to oxidative stress and mitochondrial dysfunction is influenced by host factors, including genetic variants (e.g. *SOD2*, *RARG*, and *TOP2B*) and gut-derived metabolites.^70–72^ Importantly, SCFAs and bile-acid derivatives regulate cellular energy metabolism and redox homeostasis, suggesting that therapy-induced microbiome disruption may compromise systemic mitochondrial resilience, lowering the threshold for oxidative injury in cardiac and vascular tissue.^73^ Russo *et al*.^74^ demonstrated the cardioprotective effects of SCFAs by showing that exogenous administration of phenylanine-butyramide (FBA), a butyrate derivative, attenuated DOX-induced ROS production, thereby mitigating oxidative stress and mitochondrial dysfunction and preventing structural and functional cardiac damage (e.g. ventricular dilation, fibrosis, and apoptosis) in mice. These findings infer microbial metabolites, specifically SCFAs, as potential key modulators of redox balance and mitochondrial resilience in CTRCD.

### 3.2 Inflammatory, immune, and endothelial pathways

Downstream of oxidative stress and mitochondrial dysfunction, CTRCD is characterized by robust activation of inflammatory and immune signalling networks that contribute to both myocardial and vascular injury. Although most mechanistic evidence is derived from chemotherapy clinical and preclinical models (*Table 2*), emerging data suggest that gut–heart axis dysregulation represents a convergent pathway across diverse cancer therapies, primarily through shared mechanisms of barrier disruption, systemic inflammation, and endothelial injury. Oxidative stress acts as a critical link between myocardial dysfunction and vascular injury in CTRCD by upregulating redox-sensitive transcription factors, particularly nuclear factor kappa-light-chain-enhancer of activated B cells (NF-κB) and driving the expression of pro-inflammatory cytokines, including tumour necrosis factor α (TNF-α), interleukin-1β (IL-1β), and interleukin-6 (IL-6).^82^ These mediators promote cardiomyocyte injury, endothelial dysfunction, and systemic inflammatory responses. Concurrent suppression of antioxidant pathways, most notably nuclear factor erythroid 2-related factor 2 (Nrf2) signalling, further amplifies this pro-inflammatory state.^83–85^

**Table 2:** Gut–heart axis-mediated inflammatory and immune mechanisms in chemotherapy-induced chemotherapy

Mechanism/pathway	Model/population	Key findings	Outcome/relevance	Ref.
TLR4 activation via LPS translocation	Breast cancer patients (*n* = 34) treated with DOX	Chemotherapy-induced intestinal epithelial injury → systemic translocation of LPS and ↑ TNF-α levels and TLR4 expression (*P < 0.05*)	Demonstrates a mechanistic link between gut barrier dysfunction, systemic inflammation, and cardiotoxicity	^17^
Chemotherapy-induced endotoxaemia and upregulation of NOX-2 and TLR-2	DOX-treated murine model ± FMT	DOX induced intestinal injury, increasing gut permeability and circulating LPS levels, alongside upregulated cardiac NOX-2 and TLR-2 expression. FMT restored gut barrier integrity, reduced endotoxaemia, significantly downregulated NOX-2 and TLR-2 expression and improved cardiac function	Provides causal evidence that chemotherapy-induced disruptions to the gut microbiome contribute to cardiotoxicity via systemic endotoxaemia and downstream inflammatory signalling	^75^
TLR4 deletion	DOX-treated murine model	TLR4^−/−^ mice exhibited attenuation of DOX-induced cardiomyopathy, reduced myocardial inflammation, and improved LV function (*P < 0.05*)	Confirms a causal role of TLR4-mediated signalling in the development of cardiotoxicity	^76^
TLR2 and TLR4 upregulation in cardiomyocytes	Breast cancer patients (*n* = 25) treated with DOX	DOX treatment upregulated TLR2 and TLR4 expression in myocardial tissue, correlating with clinical indicators of cardiotoxicity. Elevated baseline serum TLR4 levels predicted increased risk of cardiotoxicity	Identifies TLR4 as a potential prognostic biomarker for chemotherapy-related cardiac injury	^77^
Differential expression of TLR2 and TLR3	Patients with DOX-induced heart failure vs. healthy, HF, and DOX-only controls (*n* = 236)	TLR2 expression was significantly elevated, while TLR3 expression was markedly reduced in DOX + HF patients (both *P < 0.02*)	Suggests divergent immunomodulatory roles of TLR2 (pro-inflammatory) and TLR3 (protective) in chemotherapy-induced cardiotoxicity pathogenesis	^78^
TLR2 deletion	DOX-treated murine model	TLR4^−/−^ mice demonstrated suppressed NF-κB activation, reduced pro-inflammatory cytokine production (*P < 0.01*), and improved survival following DOX treatment (46% vs. 11%, *P < 0.05*)	TLR2-mediated inflammation as a critical driver of DOX-induced myocardial injury	^79^
Endothelial inflammation via ICAM-1	Human endothelial cells exposed to cisplatin	Cisplatin exposure upregulated ICAM-1 expression via NF-κB activation, promoting leucocyte adhesion and vascular inflammation. ICAM-1 blockade mitigated these effects	Highlights endothelial activation as a mechanism of vascular toxicity in chemotherapy-induced cardiotoxicity	^80^
MAPK and NF-κB activation secondary to mucositis	5-FU-treated murine model	5-FU-induced mucositis activated MAPK and NF-κB signalling, leading to systemic cytokine release and inflammation	Demonstrates that gut injury-driven inflammation contributes to vascular and myocardial toxicity	^81^

TLR4, toll-like receptor 4; DOX, doxorubicin; FMT, faecal microbiota transplantation; LPS, lipopolysaccharide; LV, left ventricular; HF, heart failure, ICAM-1, intercellular adhesion molecule 1.

Endothelial dysfunction is a central feature across multiple therapies and is characterized by impaired endothelial nitric oxide synthase (eNOS) activity and reduced NO bioavailability.^86,87^ This imbalances promotes endothelial oxidative injury and vascular dysfunction, leading to the increased expression of adhesion molecules (ICAM-1, VCAM-1), facilitating leucocyte recruitment, vascular inflammation, and increased endothelial permeability.^61,87–89^ Sustained endothelial disruptions extend to underlying vascular layers, stimulating vascular smooth muscle cell (VSMC) activation, extracellular matrix remodelling, and vascular fibrosis.^69^ Activation of the renin–angiotensin–aldosterone system (RAAS), particularly angiotensin II signalling through angiotensin II receptor type 1 (ATR1), further amplifies inflammatory and vascular dysfunction.^90–92^

The gut microbiome serves as an upstream amplifier of these inflammatory pathways and endothelial injury. Therapy-induced disruption increases intestinal permeability, facilitating systemic translocation of microbial components, such as LPS, which activates toll-like receptor 4 (TLR4) signalling in cardiovascular and immune cells.^11^ Concurrent alterations in SCFA signalling via receptors, including G-protein-coupled receptors (GPR41 and GPR43), further modulate immune and endothelial responses^93^ while amplifying systemic inflammatory cascades.^94,95^ Depletion of SCFA-producing genera, including *Bacillus* and *Actinomyces*, has been documented following cancer therapy and correlates with increased intestinal permeability, elevated circulating LPS and microbial translocation, and heightened systemic inflammation.^96^

### 3.3 Apoptosis and necrosis of cardiomyocytes

Cardiomyocyte loss represents the final common downstream consequence of the integrated metabolic inflammatory and vascular disturbances described above.^97,98^ Classical mechanisms include apoptosis and necrosis mediated through mitochondrial pathways. Excessive ROS promotes opening of mitochondrial permeability transition pores (mPTPs) and release of cytochrome-c, triggering caspase activation and apoptotic cell death.^98–100^ When cellular injury exceeds apoptotic capacity, necrosis occurs, resulting in the release of damage-associated molecular patterns (DAMPs) that further amplify inflammatory signalling and myocardial injury.^61^

Several cancer therapies induce immunogenic forms of cell death, resulting in the release of DAMPs. In the setting of cancer therapy-induced microbiome dysbiosis where intestinal barrier function is compromised, the combined systemic exposure to DAMPs and translocated LPS amplifies TLR-mediated inflammatory signalling. In particular, preclinical anthracycline models demonstrate that chemotherapy-induced alterations in gut microbial composition increase systemic LPS exposure, thereby exacerbating vascular inflammation and myocardial injury through oxidative and NF-κB-mediated pathways. Notably, faecal microbiota transplantation (FMT) in doxorubicin (DOX)-treated mice improved both intestinal barrier disruption and cardiovascular dysfunction, suggesting a potential mechanistic contribution of gut microbiome dysregulation in CTRCD.^76^ Additionally, preclinical studies on cultured rat embryonic ventricular myocardial H9c2 cells treated showed that DOX induced AMP-activated protein kinase (AMPK) activation, which upregulated to apoptotic pathways.^101^

More recently, additional regulated cell death pathways have been implicated. These include ferroptosis, an iron-dependent lipid peroxidation process; necroptosis, mediated by receptor-interacting protein kinase 1/3 (RIPK1/3) signalling; pyroptosis, an inflammatory form of cell death associated with inflammasome activation; and dysregulated autophagy, a double-edged sword in cardiovascular health.^100,102–106^ These processes are particularly relevant in chemotherapy-induced cardiotoxicity but may also extend to other therapies through shared inflammatory and metabolic pathways. Epigenetic regulators, including microRNAs and chromatin-modifying mechanisms, further modulate these responses and may contribute to interindividual variability in cardiotoxic susceptibility.^107^

## 4. The role of microbial metabolites in CTRCD

The gut microbiome plays a critical role in cardiovascular physiology and disease.^11,108,109^ As highlighted by the link between the gut–heart axis and the pathology of CTRCD, central to this interplay are microbially derived metabolites that regulate gut barrier integrity, immune signalling, and vascular homeostasis.^11^ Disruptions to microbial composition and subsequent metabolite production are associated with oxidative stress, endothelial dysfunction, and inflammatory cascades and may therefore contribute to or further compound cardiovascular risk.^110–113^ Here, we highlight the cardioprotective effects of SCFAs, pro-inflammatory effects of LPS and TMAO, and dual functionality of gut-derived secondary bile acids. Importantly, the role of microbial metabolites in CTRCD carries clinical significance given these may serve as reliable biomarkers that capture upstream processes not reflected by conventional biomarkers. Current biomarkers used for CTRCD, including cardiac troponins and natriuretic peptides, primarily indicate cardiomyocyte injury and haemodynamic stress, while imaging-based parameters detect subclinical functional impairment.^114–117^ In contrast, levels of microbial metabolites may reflect gut barrier dysfunction and systemic inflammatory priming, potentially preceding detectable myocardial injury.

### 4.1 Cardioprotective effects of short-chain fatty acids

SCFAs, primarily acetate, propionate, and butyrate, are microbial metabolites generated through fermentation of dietary fibre by commensal gut bacteria. They are key mediators of gut–heart communication, influencing intestinal integrity, immune regulation, and inflammatory signalling with broad cardioprotective effects.^118–120^ Among SCFAs, butyrate plays a crucial role in intestinal homeostasis, serving as the primary energy source of colonocytes.^121^ By supporting colonocyte energy metabolism, butyrate maintains epithelial barrier integrity and regulates mucosal immune responses, thereby limiting microbial translocation and systemic exposure to pro-inflammatory gut-derived components.^122,123^ Preclinical models show that butyrate supplementation improves intestinal permeability and reduces circulating LPS levels, by up to 24%, leading to attenuated systemic inflammation and decreased cardiovascular stress.^124^

**Figure 2:**
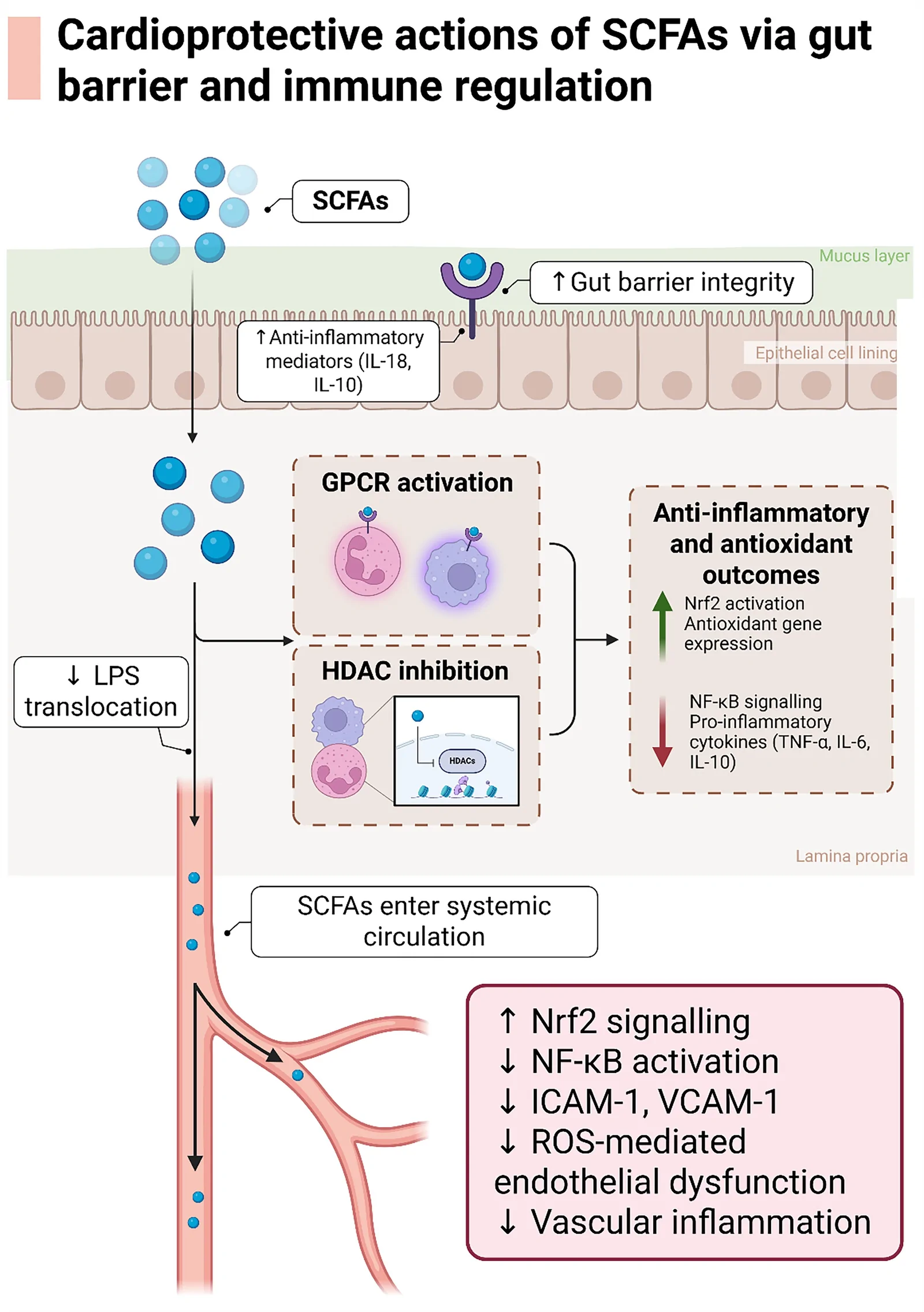
Cardioprotective actions of SCFAs via gut barrier and immune regulation. The role of SCFAs in regulating gut barrier integrity and reducing the translocation of LPS, as well as modulating innate immune cells through parallel activation of GPCR-dependent signalling and inhibition of HDACs. These pathways converge to suppress NF-κB activity and enhance Nrf2-mediated antioxidant responses, limiting release of pro-inflammatory cytokines. Moreover, upon entering systemic circulation, endothelial dysfunction and vascular inflammation are further reduced by SCFAs, thereby protecting against inflammation-driven cardiovascular pathology.

Beyond sustaining epithelial homeostasis, SCFAs exert potent immunomodulatory effects through both receptor-mediated and epigenetic mechanisms (summarized in *Figure 2*). They act as ligands for GPCRs, including free fatty acid receptors (FFAR2 and FFAR3), expressed on immune and ECs, regulating leucocyte recruitment, cytokine production, and inflammatory tone.^125–128^ In parallel, butyrate and propionate function as histone deacetylase (HDAC) inhibitors, promoting anti-inflammatory gene expression and influencing immune cell differentiation.^129,130^ SCFA signalling through GPR41 and GPR43 on immune cells is essential for maintaining gut barrier integrity and restraining pro-inflammatory TLR4 activation.^131^ Mice lacking both receptors exhibit increased macrophage infiltration, heightened gut permeability with systemic LPS translocation, and worsened hypertensive and fibrotic responses, providing direct evidence that SCFA-immune crosstalk is central to blood pressure regulation and cardio-protection.^93^ Through HDAC inhibition, butyrate enhances histone acetylation and activates transcription factors, such as Nrf2 and inhibits NF-κB, pathways central to oxidative stress regulation and endothelial function.^132^ Nrf2 activation, in particular, upregulates antioxidant proteins that mitigate oxidative injury in myocardial tissue.^133–135^

Critically, cancer therapies impair SCFA production through depletion of fibre-fermenting taxa, presenting a key mechanistic link between gut dysregulation, microbial ecology, and cardiovascular health.^21^ Disruptions to SCFA biosynthesis and metabolism have been consistently associated with adverse cardiovascular outcomes. Individuals with heart failure, atherosclerosis, and vascular dysfunction frequently exhibit reduced abundance of butyrate-producing bacteria, suggesting a loss of protective microbial function in cardiovascular pathology.^118–120^ Recent meta-analyses reported that heart failure patients exhibit markedly reduced gut microbial diversity, elevated *Streptococcus* and *Bacteroides* abundance, disrupted SCFA production, and increased levels of TMAO-producing bacteria.^113^ Similarly, Yang *et al.*  ^136^ (*n* = 371) found that individuals enriched in *Faecalibacterium prausnitzii* had the lowest incidence of coronary artery disease, consistent with preclinical findings showing that *F. prausnitzii* supplementation attenuated atherosclerotic plaque formation and inflammation in apolipoprotein E-deficient (ApoE^−/−^) mice. SCFAs additionally influence host lipid metabolism and vascular homeostasis, further reinforcing their cardioprotective profile.^137^

Mechanistically, SCFAs can modulate intestinal epithelial AMPK signalling, suppressing microbial enzymatic pathways involved in trimethylamine (TMA) production and thereby reducing hepatic synthesis of the pro-atherogenic metabolite TMAO.^138^ This suppression can reduce TMAO synthesis by up to 62.6%, associated with lower circulating lipid and cholesterol levels and attenuated atherosclerotic risk.^139^ In a cohort study (*n* = 33), reduced butyrate levels were associated with increased risk of abdominal aortic aneurysm development, underscoring the translational relevance of SCFA deficiency.^140^ In preclinical hypertension models, high-fibre diets or acetate supplementation restored gut microbial balance, increased SCFA-producing taxa (notably *Bacteroides acidifaciens*), lowered systolic and diastolic blood pressure, and reduced cardiac and renal fibrosis in hypertensive mice.^12^

### 4.2 Pro-inflammatory effects of LPS and TMAO

In contrast to SCFAs, gut-derived LPS and TMAO act as potent pro-inflammatory mediators that link a dysregulated gut microbiome to cardiotoxic outcomes. Elevated circulating levels of these endotoxins are a hallmark of cardiovascular injury that has been associated with various cardiovascular complications, including hypertension, chronic heart failure, myocardial infarction, and atherosclerosis.^141–143^ In cardio-oncology, cancer therapies can dysregulate the homeostatic state of the gut microbiome increasing the translocation of LPS and TMAO into circulation, appearing to amplify myocardial and vascular injury, contributing to the development and progression of CTRCD.

#### 4.2.1 LPS: gut barrier translocation, TLR activation, and cardiovascular implications

LPS is a gut microbiome-derived endotoxin, forming a structural component of Gram-negative bacterial outer membranes. Under conditions of barrier dysfunction, LPS translocates into systemic circulation, where it activates inflammatory pathways via TLRs (i.e. TLR4), stimulating the release of pro-inflammatory cytokines and contributing to endothelial dysfunction, oxidative stress, and cardiac injury.^144,145^

Clinical evidence strongly supports a link between heightened systemic LPS levels and adverse cardiovascular outcomes. In a prospective cohort of patients with atrial fibrillation (*n* = 912), Pastori *et al.*^18^ demonstrated that there was a significant association between elevated circulating LPS levels and the incidence of major adverse cardiovascular events (MACEs). Similarly, a longitudinal study by Asada *et al.*^146^ (*n* = 2568) reported that higher serum levels of LPS-binding protein, produced in response to LPS microbial translocation reflecting systemic endotoxaemia, were associated with an increased risk of incident CVD, defined as the first occurrence of stroke or coronary heart disease.

**Figure 3:**
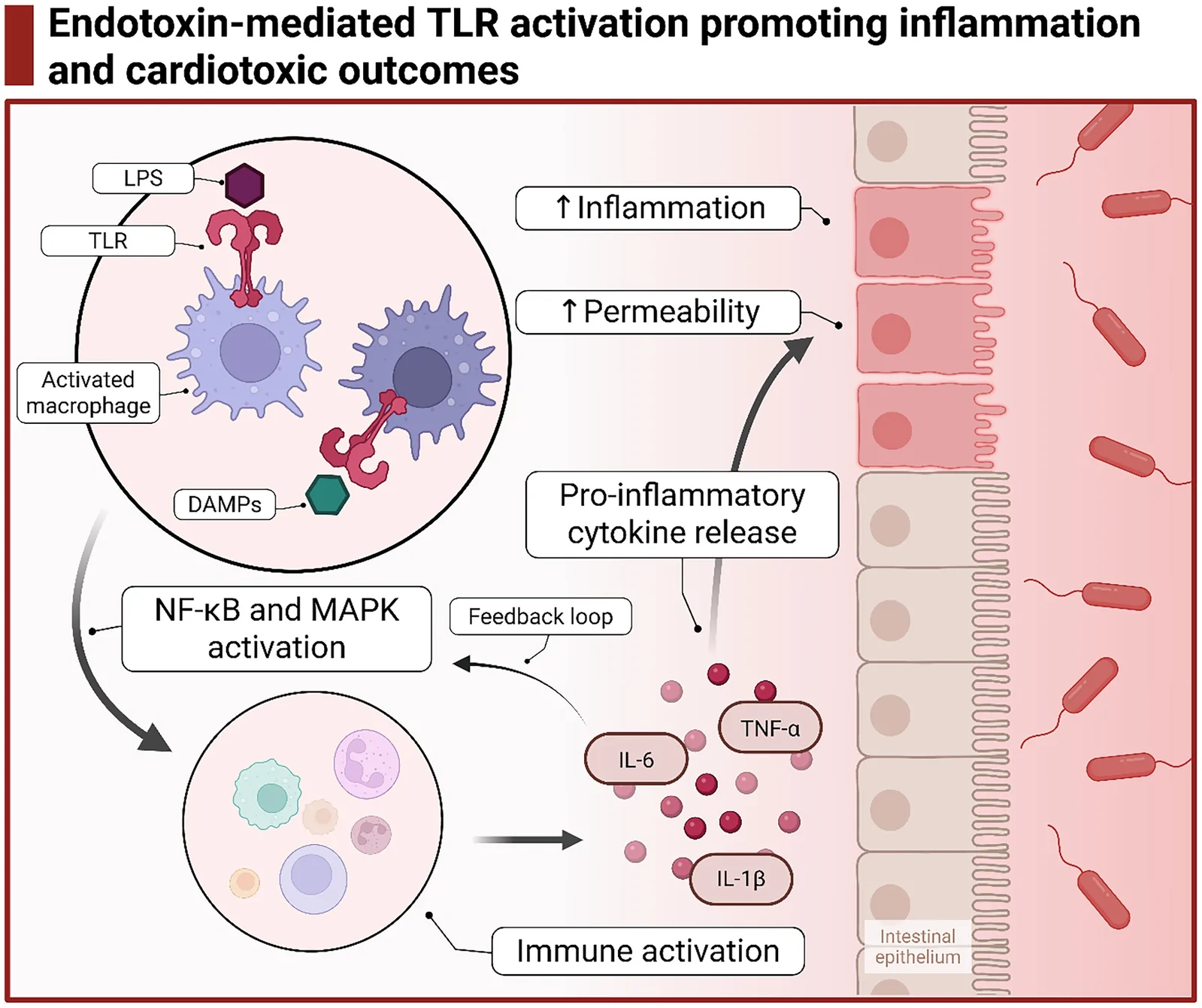
Endotoxin-mediated TLR activation promoting inflammation and cardiotoxic outcomes. TLR activation through the upregulation of DAMPs and LPS, leading to NF-κB and MAPK pathway activation, and release of pro-inflammatory cytokines. This inflammatory response is further reinforced by a feedback loop, resulting in increased permeability of the gut barrier integrity, promoting systemic inflammation and cardiotoxic outcomes.

Once in circulation, LPS binds to LPS-binding protein and CD14 on monocytes, macrophages, and ECs, forming a complex that activates TLRs.^145^ This engagement with TLRs triggers a pro-inflammatory cascade via the NF-κB pathway, as well as adhesion molecules, such as ICAM-1, that promote leucocyte recruitment to the vascular wall.^80^ This results in increased transcription of pro-inflammatory cytokines, which establishes a feedback loop amplifying systemic inflammation and dysregulation of intestinal endothelial and vascular barrier function, leading to the release of DAMPs.^147^ Importantly, this pathway is not limited to chemotherapy but may also be triggered by radiotherapy and ICIs through mucosal injury and immune activation.^59^ Therefore, gut barrier disruption, TLR activation, and cytokine release form a core pathway linking therapy-induced intestinal injury to cardiotoxic outcomes, as summarized in *Figure 3*.

#### 4.2.2 TMAO: vascular inflammatory signalling and cardiotoxic outcomes

TMAO is a gut microbiota-derived metabolite generated through the microbial metabolism of dietary nutrients, including L-carnitine, choline, and betaine into TMA, which is subsequently oxidized to TMAO within the liver.^148^ Importantly, circulating TMAO levels are not solely determined by microbial production but are also strongly influenced by renal clearance, as TMAO is primarily excreted via the kidneys.^149^ This is particularly relevant in oncology populations, where chemotherapy-induced nephrotoxicity, altered hydration status, and comorbidities may compromise renal function, potentially confounding interpretation of TMAO as a biomarker of microbiome activity or cardiovascular risk.^150^ This metabolite has been implicated in inflammatory modulation with the expansion of TMA-producing taxa, such as *Streptococcus*, associated with a dysregulated gut microbiome and increases in TMAO production, promoting a pro-atherogenic state.^148,151,152^

In contrast to LPS, TMAO does not function as a classical endotoxin by engaging in pattern recognition receptors directly. Instead, TMAO promotes vascular inflammation through metabolite-driven intracellular signalling pathways.^153^ TMAO exacerbates vascular oxidative stress by increasing ROS generation and impaired NO bioavailability, thereby contributing to endothelial dysfunction and vascular injury.^154^ Preclinical studies further demonstrate that TMAO activates the NLRP3 inflammasome, leading to the activation of caspase-1 and downstream release of pro-inflammatory cytokines (i.e. IL-1β, IL-18, and IL-6) to induce vascular inflammation.^19^ In parallel, TMAO has been shown to activate NF-κB and MAPK signalling pathways, amplifying transcriptional processes associated with inflammation, including inflammasome-driven inflammation and endothelial activation.^153^ Collectively, these processes establish TMAO as a metabolite-mediated inflammatory amplifier that complements endotoxin-driven signalling to promote vascular dysfunction and cardiovascular pathology.

Multiple studies further implicate TMAO in CVD severity and adverse outcomes, as patients with heart failure and increased CVD severity exhibit higher TMAO, choline, and betaine levels compared to healthy individuals.^155–158^ For example, Makrecka-Kuka *et al.*^159^ (*n* = 22) demonstrated that elevated TMAO levels impaired cardiac mitochondrial function by reducing both pyruvate and fatty acid oxidation, thereby promoting development of CVD in a preclinical model. Additionally, in a large prospective cohort study (*n* = 4007), Tang *et al.*^156^ showed that elevated baseline TMAO levels were strongly associated with MACE. Similarly, Suzuki *et al*. identified TMAO as an independent predictor of 2-year mortality following acute myocardial infarction (*n* = 1079), supporting its potential as a biomarker for cardiovascular risk.^14,160^ Consistent with these findings, elevated serum TMAO levels were strongly associated with impaired endothelial function in patients with hypertension (*n* = 110).^161^

### 4.3 Bile acids as dual modulators of cardiometabolic function

Gut-derived bile acids are also critical regulators of cardiometabolic host health, acting as both signalling molecules and metabolic modulators. Unlike SCFAs or microbial endotoxins, bile acids exert their effects through host nuclear and membrane receptors, influencing oxidative stress, inflammation, lipid metabolism, and cardiac function.^162^ Emerging evidence suggests that alterations in bile acid composition and signalling may contribute to both protective and deleterious cardiovascular outcomes, positioning bile acids as key mediators within the gut–heart axis.

Primary bile acids, including cholic acid and chenodeoxycholic acid (CDCA), are cholesterol-derived metabolites synthesized in the liver and metabolized by the gut microbiome into secondary bile acids, such as deoxycholic acid (DCA), ursocholic acid (UCA), ursodeoxycholic acid (UDCA), and lithocholic acid (LCA).^163–165^ As ligands for various nuclear receptors, including nuclear farnesoid X receptor (FXR) and the membrane bound G-protein-coupled bile receptor 1 (TGR5), bile acids circulate systemically and regulate metabolic pathways, lipid profiles, and immune signalling.^162^

Activation of FXR in the liver and vasculature modulates cholesterol and triglyceride metabolism, promotes anti-inflammatory gene expression, and regulates oxidative stress, while TGR5 signalling in ECs and immune populations can enhance NO bioavailability, attenuate pro-inflammatory cytokine production, and improve vascular tone.^166^ Dysregulation of bile acid signalling, whether through altered hepatic synthesis, microbial disruption, or receptor expression, has been associated with endothelial dysfunction, impaired lipid handling, and increased cardiovascular risk.^167^

Cancer therapy-induced alterations in bile acid composition may similarly contribute to cardiotoxicity by promoting oxidative stress, mitochondrial dysfunction, and overall cellular damage.^162^ The biologic effects of bile acids are influenced by their structure and hydrophobicity, and this pool is often shifted towards more hydrophobic and pro-inflammatory secondary bile acids during gut microbial dysregulation. For instance, hydrophobic bile acids (e.g. LCA, DCA, and CDCA) exert cardiotoxic effects, with elevated levels impairing mitochondrial dysfunction, contributing to cardiac dysfunction.^15,168,169^ Elevated DCA levels, for example, have also been associated with increased risk of MACE through NF-κB activation.

In contrast, hydrophilic bile acids, such as UDCA, have demonstrated cardioprotective properties.^168–171^ Preclinical studies demonstrate that UDCA administration reduces myocardial damage by counteracting the membrane-damaging effects of hydrophobic bile acids.^172^ Clinically, UDCA treatment in patients with chronic heart failure (*n* = 17) improved ischaemic blood flow and lowered levels of TNF-α receptor, suggestive of reduced systemic inflammation and improved myocardial injury.^169,173^ As chemotherapy disrupts the balance between hydrophobic and hydrophilic bile acids, the cardioprotective mechanisms of these metabolites are weakened, increasing vulnerability to cardiotoxicity.

## 5. Knowledge gaps and limitations: defining the gut microbiome in cardio-oncology

While increasing evidence supports the link between the gut microbiome and CTRCD via the gut–heart axis, further work is critically required to improve our understanding of the mechanisms and clinical implications (*Table 3*). Most importantly, it is essential to distinguish that much of the current evidence linking the gut–heart axis to CTRCD remains associative, with direct causal relationships largely inferred from preclinical models. While experimental studies suggest that modulation of microbial pathways can influence pathways relevant to CTRCD, clinical evidence establishing a definitive gut–heart causal axis in this setting remains limited. This is largely due to the relative infancy of this field, demanding further, strategically designed preclinical and clinical assessments that directly test causality of the gut microbiome in CTRCD. Further, the major portion of the knowledge base centres around the known mechanisms of chemotherapy on both the gut microbiome and cardiac function. This suggests that key knowledge gaps exist relating to the effects of other cancer therapies, including immunotherapies, targeted therapies, and radiation on the gut–heart axis in the context of CTRCD.

**Table 3:** Key knowledge gaps and future directions for understanding the role of the gut–heart axis in CTRCD

Key considerations	Limitations/knowledge gaps	Research priorities
Treatment modalities	Limited understanding role of the gut–heart axis beyond conventional chemotherapy (e.g. immunotherapy, targeted agents, and radiotherapy).	Conduct preclinical and clinical studies to elucidate the role of the gut microbiome in emerging treatments within cardio-oncology. Define microbiome–drug interactions.
Temporal dynamics	Microbiome alterations that occur during therapy may precede cardiac injury. Temporal relationship between dysbiosis and CTRCD is poorly defined. Limited understanding of the role of the gut–heart axis in acute vs. chronic CTRCD.	Conduct prospective longitudinal clinical studies integrating microbiome, metabolomic, and comprehensive cardiac phenotyping data. Define temporal dynamics linking gut dysbiosis with cardiotoxic endpoints. Develop early earning biomarkers (e.g. microbial metabolites preceding troponin increase).
Causality vs. association	Preclinical studies indicate microbiome modulation can influence cardiac outcomes. Evidence is largely preliminary and associative. It remains unclear whether microbial metabolites as biomarkers reflect causal mechanisms or systemic toxicity.	Perform longitudinal clinical studies integrating microbiome profiling with cardiac phenotyping. Undertake mechanistic investigations to directly link chemotherapy-induced microbiome alterations with cardiovascular outcomes in humans.
Interindividual variability/confounding factors	Microbial composition is influenced by multiple interacting host factors (i.e. age, sex, genetics, renal function, and cancer-associated cachexia). Treatment-related variables (i.e. modality, cumulative dose, and concurrent cardioprotective medications) directly alter both microbiome composition and cardiotoxicity risk, complicating causal inference. Lifestyle and environmental factors (i.e. dietary fibre/fat intake, smoking, alcohol consumption, and physical activity) strongly modulate microbial diversity and metabolite protection, potentially overshadowing therapy-related effects. Methodological variability (16S vs. metagenomic sequencing, batch effects and stool sampling timing) introduces additional heterogeneity across studies. Confounding factors may mask true associations.	Standardize study protocols incorporating comprehensive metadata collection (diet, medications, and comorbidities). Implement stratified analyses accounting for host clinical variables and treatment heterogeneity. Harmonize microbiome sequencing and metabolomic pipelines to reduce technical variability. Validate findings across multiple, well-characterized cohorts.
Reproducibility across studies	Microbial biomarkers show promise in preclinical and small clinical studies. Heterogeneity in cancer types, treatments, patient populations, and analytic methodologies limits reproducibility.	Standardize microbiome sampling, sequencing, and metabolomic analytic protocols. Implement harmonized multi-centre studies to improve cross-cohort reproducibility. Develop reference standards and establish robust, clinically validated biomarkers suitable for routine use.
Integration into precision medicine	Current frameworks are largely reactive. Integration with other omics data has not been established.	Integrate microbiome data with genomic, metabolomic, clinical, and imaging datasets. Develop multi-omics models for risk stratification and early intervention. Use integrated models to guide chemotherapy optimization and microbiome-targeted co-therapies.
Therapeutic potential	Clinical efficacy of microbiome-targeted strategies remains unestablished. Translation to standard care has not been tested.	Conduct randomized controlled trials evaluating microbiome-targeted interventions alongside chemotherapy. Undertake translational studies to define implementation pathways in cardio-oncology.
Biomarker development	Lack of standardized, clinically validated microbiome-derived biomarkers. Unclear thresholds and clinical cut-offs.	Identify and validate circulating and stool-based biomarkers (e.g. TMAO, SCFAs, and bile acids). Establish reference ranges and clinical thresholds to enable non-invasive screening tools for cardiotoxicity that complements existing biomarkers (troponin, NT-pro-BNP).
Clinical trial design	Cardio-oncology trials rarely incorporate/consider the role of the gut microbiome Lack of standard endpoints linking microbiome to CTRCD.	Embed microbiome endpoints into oncology and cardio-oncology clinical trials. Standardize CTRCD definitions and microbiome outcomes to improve comparability across trials.
Implementation and feasibility	Limited infrastructure for microbiome testing in clinical practice. Cost, turnaround time, and regulatory barriers.	Develop scalable, cost-effective assays to enable real-world adoption in cardio-oncology clinics. Define clinical workflows and decision algorithms.

To truly advance the field forwards, effective monitoring of both acute and chronic CTRCD requires improvement. Assessing CTRCD remains a critical challenge in cardio-oncology, as early detection is essential to prevent irreversible cardiovascular injury and optimize cancer treatment outcomes. Current clinical monitoring strategies rely on circulating biomarkers, such as cardiac troponins and natriuretic peptides, alongside imaging-based measures, including LV ejection fraction and global longitudinal strain. However, these modalities primarily detect established or functional myocardial injury, limiting their sensitivity for early subclinical changes and interindividual risk stratification prior to overt dysfunction. In contrast, microbiome-derived metabolites (e.g. TMAO and SCFAs) and compositional signatures may reflect upstream host–environmental interactions that precede measurable myocardial injury, positioning them as potential complementary early-risk biomarkers rather than replacements for established cardiac diagnostics. We propose that addressing the key knowledge gaps identified and detailed in *Table 3* will position the gut microbiome as a potential novel diagnostic and therapeutic target, offering opportunities for earlier detection, risk stratification, and mitigation of cardiotoxicity.

Addressing key knowledge gaps surrounding the gut–heart axis in CTRCD through rigorous, mechanistically informed research will be essential to establish causality and temporal dynamics. This will enable integration of the gut microbiome as a complementary component to current monitoring strategies, which remain inherently limited. Microbial-derived signatures offer the potential to capture upstream processes, including inflammation, endothelial dysfunction, and metabolic dysregulation, that precede overt cardiovascular injury. However, realizing this potential will require overcoming important challenges to enable the development of robust predictive biomarkers and targeted therapeutic strategies, positioning microbiome modulation as an adjunctive approach to improve cardiotoxicity prevention and management.

## 6. Conclusion

This review highlights the emerging, yet incompletely defined, role of the gut–heart axis in modulating cardiovascular outcomes during cancer treatment. Growing evidence implicates the gut microbiome and its metabolites, including SCFAs, bile acids, TMAO, and LPS, in key pathological processes, such as inflammation, oxidative stress, and cardiac and vascular injury. However, current evidence remains largely associative, with limited mechanistic and clinical validation.

Microbial composition and microbial-derived molecules are suggested to provide novel biomarkers to assess patient susceptibility, while metabolites, such as SCFAs and hydrophilic secondary bile acids, show promise in reducing inflammation and improving cardiac resilience. Nonetheless, mechanistic insights remain incomplete, and the translation of preclinical findings into clinical application is still limited. Future studies integrating multi-omics approaches with functional validation will be essential to define casual pathways and develop microbiome-targeted strategies that prevent and mitigate cardiotoxicity, ultimately improving long-term outcomes for cancer survivors.

## Data Availability

No new data were generated or analysed in support of this research.
